# Monocyte‐Differentiation‐Activated Fluorescent “Scout” Probe for Precise in Vivo Detection of Vulnerable Plaque

**DOI:** 10.1002/advs.202515289

**Published:** 2025-12-02

**Authors:** Zechuan Li, Jiankai Dong, Zhengkun Liu, Chaoke Zhang, Jisen Li, Ying Tao, Ding Yang, Yansong Liu, Haoting Chen, Lu Liu, Jingsen Ji, Feng Cao, Dan Ding, Qian Liu, Chenxing Fu, Weisheng Guo

**Affiliations:** ^1^ Department of Cardiology Guangzhou Institute of Cardiovascular Disease Guangdong Key Laboratory of Vascular Diseases The Second Affiliated Hospital School of Biomedical Engineering Guangzhou Medical University Guangzhou 510260 China; ^2^ Department of Urology Tianjin First Central Hospital Tianjin 300192 China; ^3^ Department of Cardiology The First Affiliated Hospital of Bengbu Medical University Bengbu 233000 China; ^4^ Key Laboratory of Bioactive Materials for the Ministry of Education College of Life Sciences Nankai University Tianjin 300071 China; ^5^ Department of Radiology Peking University Cancer Hospital & Institute Key Laboratory of Carcinogenesis and Translational Research (Ministry of Education/Beijing) Beijing 100142 China; ^6^ School of Materials Science and Engineering University of Jinan Jinan 250022 China; ^7^ Nanomedicine Research Center The Third Affiliated Hospital of Sun Yat‐sen University Guangzhou 510630 China; ^8^ National Clinical Research Center for Geriatric Diseases The Second Medical Center Chinese PLA General Hospital Beijing 100853 China

**Keywords:** aggregation‐induced emission, atherosclerosis, cell‐mediated delivery, molecular imaging, monocyte dynamic differentiation

## Abstract

Precise identification of vulnerable plaque (VAP) is essential for the prevention of acute cardiovascular diseases, yet current molecular probes are hampered by poor VAP lesion penetration and high background. Here, the innate tropism of circulating inflammatory monocytes for VAP, and their differentiation‐driven expression of legumain (Lgmn) in response to the VAP microenvironment is exploited. A monocyte differentiation‐activated fluorescent (MDAF) probe is conceived that hitchhikes monocytes to precisely migrate to VAP and is activated by Lgmn during monocyte differentiation. This activation triggers in situ self‐assembly, resulting in spatiotemporally controlled aggregation‐induced emission (AIE) fluorescence signals, and turning the monocyte itself into an on‐site “scout” that reports plaque instability. In Apoe^−/−^ mice bearing both vulnerable and stable plaques, the MDAF produces a striking OFF/ON fluorescence switch confined to vulnerable lesions, yielding a markedly improved signal‐to‐noise ratio (SNR). By integrating fluorescence emission computed tomography (FLECT), MDAF probe surpasses the depth limitations of conventional fluorescence imaging. Therefore, the AIE signal of the MDAF probe is more than just a “fluorescent read‐out,” and it also acts as a crucial safety switch that transforms the monocyte into a ratiometric immune scout for plaque instability. This innovative strategy offers a translatable approach for the precision diagnosis of VAP.

## Graphical abstract

An innovative monocyte‐differentiation‐activated fluorescent “scout” probe (MDAF) leverages the inherent chemotaxis of monocytes toward vulnerable plaques (VAP) to enable efficient targeted delivery while achieving spatiotemporally controllable activation of its AIE fluorescent signals with the dynamic differentiation of monocytes, providing an intelligent, controllable imaging platform with a high signal‐to‐noise ratio (SNR) for accurate VAP diagnosis.

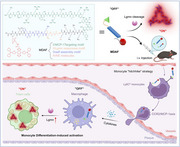

## 1 Introduction

The persistent lipid accumulation and inflammatory response in atherosclerotic areas exacerbate the formation and rupture of vulnerable plaque (VAP), significantly increasing the risk of acute cardiovascular events.^[^ ^1^, ^2^, ^3^, ^4^, ^5^ ^]^ Clinically, optical coherence tomography and intravascular ultrasound primarily assess VAP vulnerability based on pathological morphology, but these strategies fail to accurately reflect the underlying pathological processes.^[^ ^6^, ^7^, ^8^ ^]^ Although targeted molecular imaging probes or nano‐diagnostic agents enable imaging of the cellular and molecular microenvironment within VAP, their performance is limited by physiological barriers within the plaque, leading to off‐target effects and insufficient signal‐to‐noise ratio (SNR).^[^ ^9^, ^10^, ^11^, ^12^, ^13^, ^14^, ^15^, ^16^, ^17^, ^18^ ^]^ Moreover, the complexity and diversity of molecular targets and biomarkers in VAP hinder single‐target probes from comprehensively reflecting VAP vulnerability for precise diagnosis.^[^ ^19^, ^20^, ^21^ ^]^ Thus, designing intelligent and controllable probes that can fully assess VAP vulnerability holds promise for precise VAP diagnosis.

The vulnerability of plaques is intricately related to the extent of immune cell infiltration, particularly by inflammatory monocytes.^[^ ^21^ ^]^ These cells are rapidly recruited during VAP progression and differentiate into foam cells upon sensing microenvironmental cues within the plaque, leading to elevated expression of legumain (Lgmn).^[^ ^22^, ^23^, ^24^, ^25^ ^]^ This unique biological behavior positions inflammatory monocytes as ideal natural candidates for the accurate detection of VAP.^[^ ^26^, ^27^ ^]^ For instance, Leeper and colleagues leveraged inflammatory monocytes as “Trojan horses” for carbon nanotubes, enabling these nanocarriers to traverse VAP physiological barriers, restore phagocytic function, achieve efficient drug delivery, and ultimately reduce plaque burden.^[^ ^28^ ^]^ Similarly, our prior research harnessed the dynamic upregulation of Lgmn in monocytes during their differentiation into foam cells within VAP as a responsive switch to control the activation of nano‐prodrug activity.^[^ ^29^ ^]^ Collectively, these findings underscore the potential of inflammatory monocytes as natural “immune scouts” for plaque vulnerability by leveraging their inherent propensity to target and sense VAP. Specifically, their dynamic differentiation can serve as a responsive switch to regulate both drug activity and probe signal activation.

**Scheme 1:**
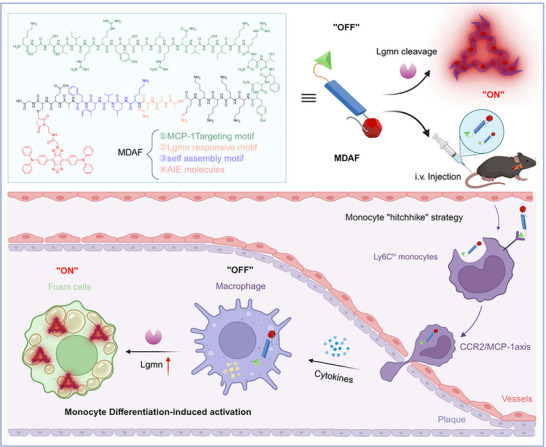
Schematic illustration of monocyte‐differentiation‐activated fluorescent probe (MDAF) for VAP detection. MDAF is capable of hitchhiking circulating inflammatory monocytes, functioning as “scouts” to achieve precise targeting at VAP lesions. Upon sensing fluctuations in Lgmn expression generated by monocyte dynamic differentiation, MDAF undergoes a responsive self‐assembly reaction to form nanofibers, thereby activating the fluorescence signal of aggregation‐induced emission (AIE) molecules and enabling accurate detection of VAP.

Inspired by the above insights, we conceived a monocyte differentiation‐activated fluorescent (MDAF) probe that leverages monocytes to precisely target VAP and is activated by legumain (Lgmn) during monocyte differentiation. As depicted in **Scheme** **1**, the MDAF probe comprises four key components, including an MCP‐1 targeting motif (KSSTIRRYSALRQVSIKRNTFNY), an Lgmn‐responsive cleavable motif (AAN), a self‐assembly motif (KLVFFAECG), and aggregation‐induced emission (AIE) molecules. Our results validate that MDAF efficiently targets monocytes by integrating the MCP‐1 targeting motif, as evidenced by in vitro uptake assays and in vivo flow cytometric analysis. Upon differentiation of monocytes into foam cells with increased Lgmn expression, the MDAF probe undergoes specific recognition and cleavage at the AAN motif, which triggers self‐assembly into nanofibers and activates AIE fluorescence for precise detection of VAP. With the aid of 3Dfluorescence emission computed tomography (FLECT) technology, MDAF probe can distinguish VAP from stable plaques in the same Apoe^−/−^ mice through efficient targeting and controllable signal activation. In brief, by leveraging the natural tropism of monocytes for inflamed plaques and their differentiation into foam cells, MDAF effectively converts monocytes into “scouts” that deliver a targeted and self‐amplifying fluorescence signal. This innovative approach offers a highly specific and sensitive strategy for identifying and monitoring high‐risk atherosclerotic lesions.

## 2 Results and Discussion

### 2.1 Characterization of the Lgmn‐Triggered Self‐Assembly Property of MDAF

**Figure 1:**
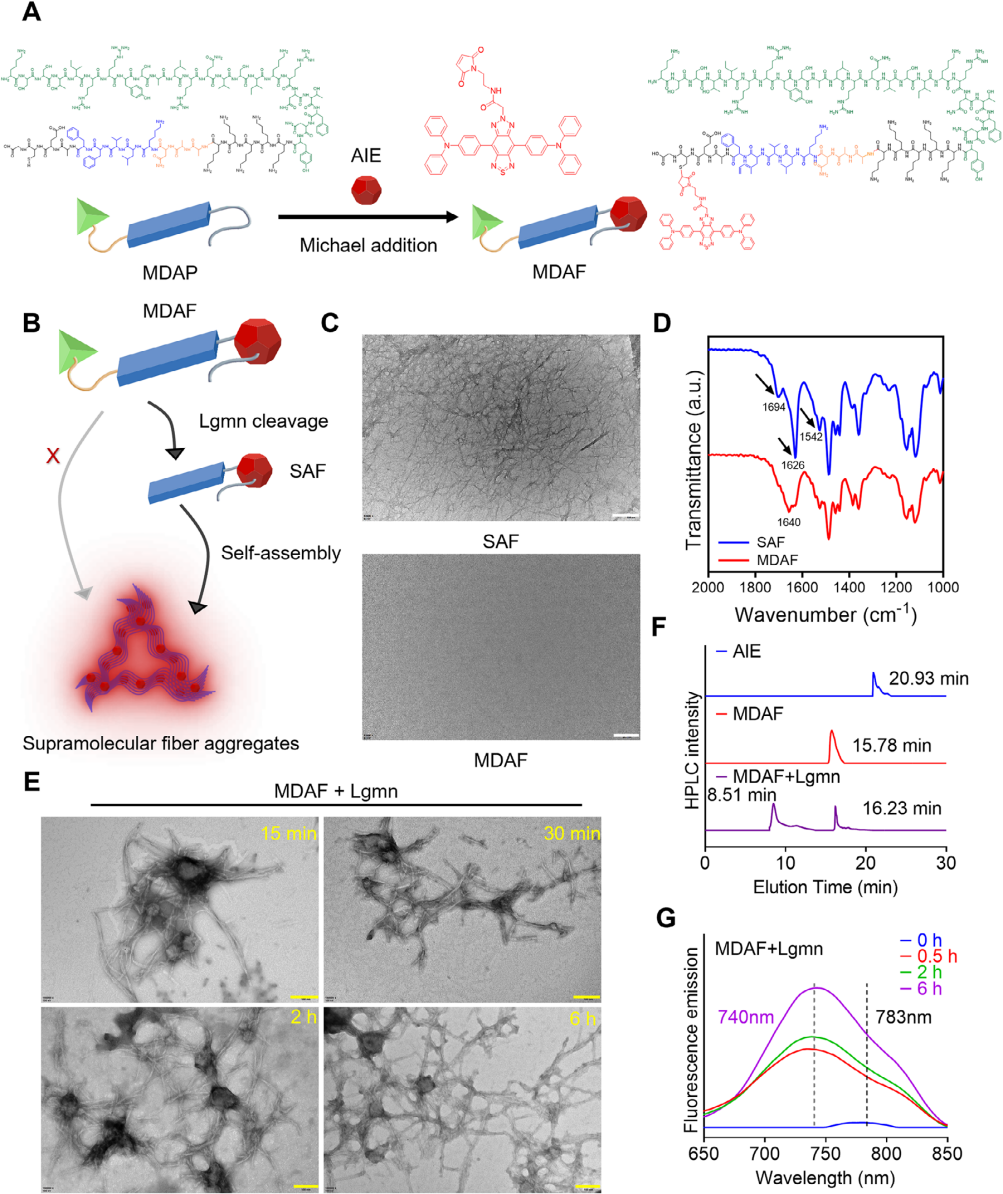
Characterization of the Lgmn‐triggered self‐assembly property of MDAF. A) Schematic diagram of the Michael addition reaction for synthesizing MDAF with MDAP and AIE molecules. B) Illustration of the activation mechanism of MDAF's fluorescence signal by enzymatically induced self‐assembly. C) TEM images of MDAF and SAF solution (1 µm). D) FTIR spectra of MDAF and SAF. E) TEM images of the self‐assembly reaction of MDAF triggered by Lgmn cleavage. F) HPLC analysis of MDAF when cleaved by Lgmn. G) Fluorescence absorption spectra of MDAF triggered by Lgmn cleavage when self‐assembling. The scale bar (white) is 200 nm, and the scale bar (yellow) is 100 nm.

To systematically characterize the Lgmn‐triggered self‐assembly property of MDAF, we synthesized three distinct peptides: MDAP (containing the MCP‐1 targeting motif, the Lgmn‐responsive motif, and the self‐assembly motif: KSSTIRRYSALRQVSIKRNTFNYKKKKKAANKLVFFAECG), NAP (targeting motif and self‐assembly motif without Lgmn‐responsive motif: KSSTIRRYSALRQVSIKRNTFNYKKKKKKLVFFAECG), and SAP (solely self‐assembly motif: KLVFFAECG) (Figure S1, Supporting Information). In addition, AIE fluorescent molecules were conjugated to these peptides via Michael addition reaction to synthesize corresponding fluorescent probes for subsequent experiments (**Figure** **1**A; Figure S2, Supporting Information). The activation mechanism of MDAF's fluorescence signal is illustrated in Figure 1B. Native MDAF is incapable of self‐assembling to activate its fluorescent signal, but after Lgmn‐mediated cleavage, the self‐assembly motif (identical to SAF) will be exposed, triggering self‐assembly into nanofibers and activating the AIE molecules’ emission. As shown in Figure 1C, MDAF does not spontaneously initiate self‐assembly to form nanofibers, whereas SAP—due to its constitutively exposed self‐assembly motif—spontaneously assembles into nanofibers. Fourier transform infrared (FTIR) spectroscopy analysis of SAP (Figure 1D) revealed an amide II band at 1542 cm^−1^, with the amide I band shifting from 1640 to 1626 cm^−1^ and a high‐energy shoulder at 1694 cm^−1^, indicative of an anti‐parallel β‐sheet conformation within SAP nanofibers. In contrast, MDAF lacks spontaneous nanofiber formation because its self‐assembly motif remains blocked.

To verify that MDAF can be cleaved by Lgmn to trigger self‐assembly, we incubated MDAF with recombinant mouse Lgmn in vitro. The results demonstrated that MDAF was activated and gradually formed nanofibers over 6 h (Figure 1E), consistent with previous findings.^[^ ^30^ ^]^ High‐performance liquid chromatography (HPLC), as shown in Figure 1F, further confirmed Lgmn‐mediated digestion, with SAP exposure validated by elution time (Figure S2E, Supporting Information). Concomitantly, AIE fluorescence was progressively activated during the self‐assembly process (Figure 1G).

### 2.2 Specific Targeting of MDAP‐Cy5.5 to Inflammatory Monocytes

**Figure 2:**
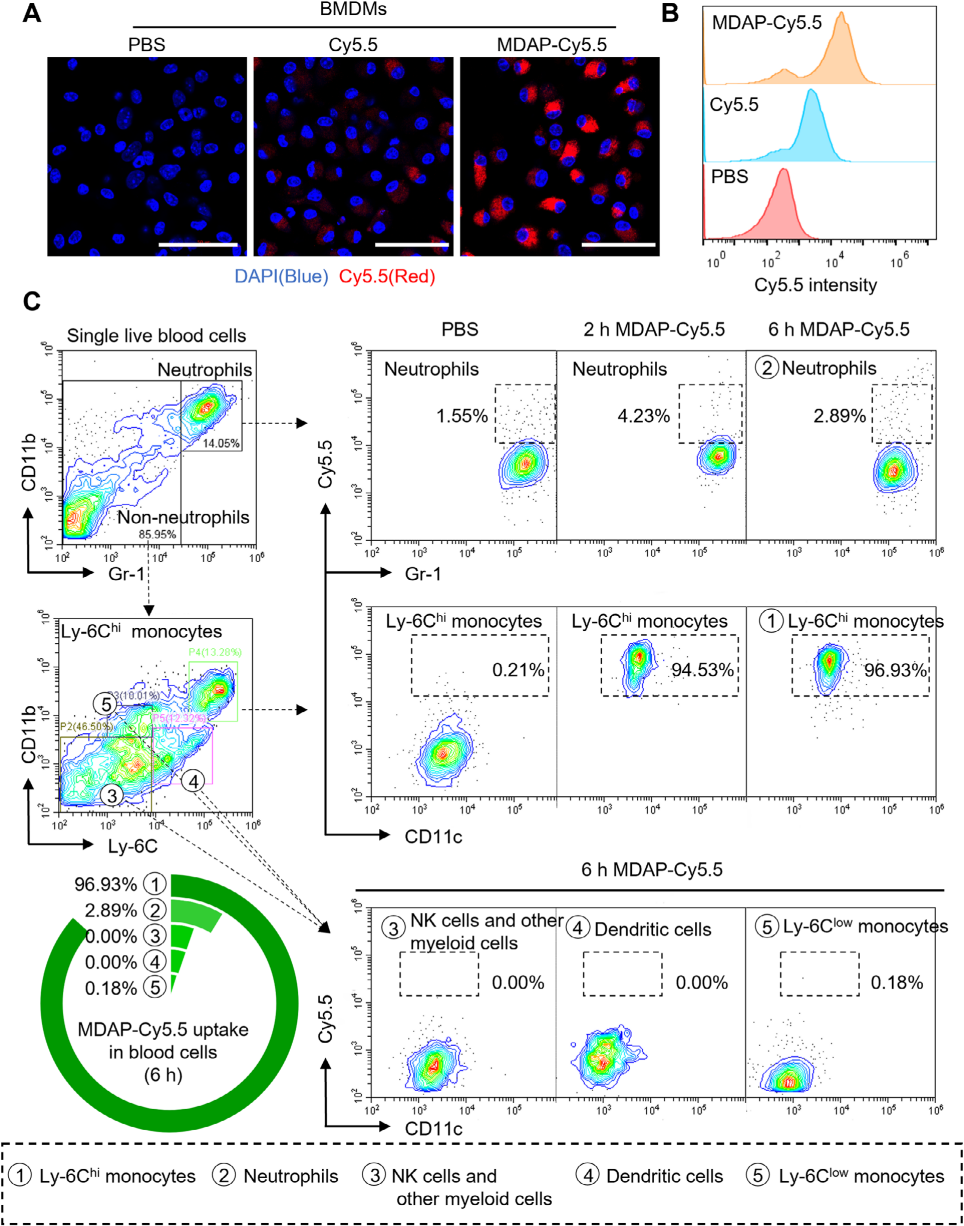
Specific targeting of MDAP‐Cy5.5 to inflammatory monocytes. A,B) Laser confocal microscopy images and flow cytometry quantitative analysis of MDAP‐Cy5.5 (Cy5.5 concentration, 5 µm) and Cy5.5 uptake by BMDMs for 1 h. (*n* = 3). The scale bar (white) is 50 µm. C) Peripheral blood mononuclear cells were collected 2 and 6 h after the administration of MDAP‐Cy5.5 (Cy5.5 dose, 0.8 mg kg^−1^) and stained with specific antibodies for flow cytometry analysis. (*n* = 3).

Following in vitro validation of MDAF's enzymatically triggered self‐assembly functions, we further evaluated its monocyte targeting ability in both in vitro and in vivo assays. Due to the unique emission characteristics of AIE molecules, the fluorescence signal of MDAF remains inactive in monocytes (lacking Lgmn expression), posing challenges for direct assessment of targeting efficiency. To address this, we substituted the AIE molecule with Cy5.5 to synthesize MDAP‐Cy5.5, a fluorescent analog designed specifically for evaluating monocyte‐targeting efficiency (Figure S3, Supporting Information). In vitro, MDAP‐Cy5.5 significantly enhanced targeting to BMDMs and RAW 264.7 cells within 1 h compared to the Cy5.5 positive control (**Figure** **2A,B**; Figure S4, Supporting Information).

We next assessed the in vivo targeting ability of MDAP‐Cy5.5 to inflammatory monocytes using Apoe^−/−^ mice. Given that nanoparticles typically undergo systemic distribution within 2–6 h (as reported in previous studies^[^ ^31^ ^]^), we selected these time points to monitor their uptake efficiency. Flow cytometry analysis of peripheral blood leukocytes (Figure 2C; Figure S5A,B, Supporting Information) revealed a cellular composition of 14.05% neutrophils and 85.95% non‐neutrophils; the latter subset was further classified into Ly‐6C^hi^ inflammatory monocytes (13.28%), Ly‐6C^low^ monocytes (12.32%), dendritic cells (10.01%), and natural killer/myeloid cells (46.50%). At 2 h post‐administration, MDAP‐Cy5.5 exhibited efficient targeting to 94.53% of circulating inflammatory monocytes, with only 5.5% uptake by other cell types. This specific targeting efficiency further increased to 96.93% at 6 h. In contrast, the Cy5.5 control showed markedly lower uptake by monocytes (36.33% at 2 h and 40.58% at 6 h; Figure S5C,D, Supporting Information), demonstrating that MDAP‐Cy5.5 enhances active targeting to inflammatory monocytes by 50–60%.

### 2.3 Monocyte Differentiation‐Responsive Activation of MDAF

**Figure 3:**
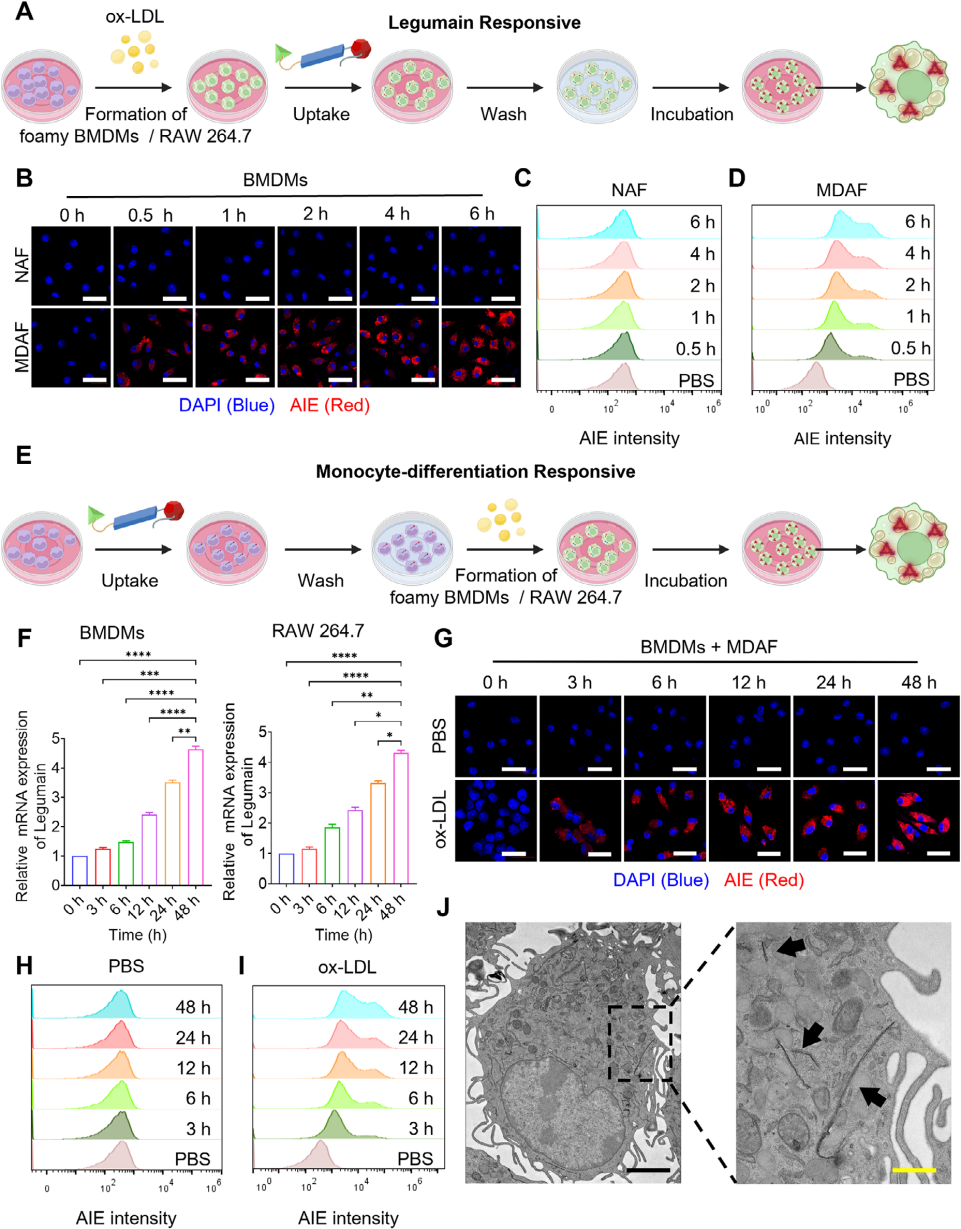
Monocyte differentiation‐responsive activation of MDAF. A) Experimental flow chart of Lgmn‐responsive activation of MDAF in foamy BMDMs and RAW 264.7 cells. B–D) Laser confocal microscopy images and flow cytometry quantitative analysis of Lgmn‐responsive activation assay of MDAF and NAF in foamy BMDMs. (*n* = 3). The scale bar (white) is 20 µm. E) Experimental flow chart of the activation of MDAF induced by the dynamic differentiation of BMDMs and RAW264.7 cells into foam cells. F) Changes of Lgmn mRNA expression in BMDMs and RAW 264.7 cells during their dynamic differentiation. G–I) Confocal laser microscopy images and flow cytometry quantitative analysis of fluorescence intensity of activated MDAF in normal and differentiating BMDMs. (*n* = 3). The scale bar (white) is 20 µm. (J) Bio‐TEM images of nanofiber assemblies formed by MDAF in foamy BMDMs. The scale bar (black) is 2 µm, and the scale bar (yellow) is 1 µm.

To verify whether MDAF undergoes Lgmn‐mediated cleavage at the cellular level and triggers self‐assembly reaction to activate its fluorescent signal, we first investigated changes in Lgmn expression during monocyte‐to‐foam cell differentiation. Notably, ox‐LDL is a key factor regulating this differentiation process. Therefore, we aim to first investigate whether varying concentrations of ox‐LDL can influence this differentiation, thereby increasing the expression of Lgmn.^[^ ^32^ ^]^ As shown in Figure S6 (Supporting Information), Lgmn expression was dependent on ox‐LDL concentration. We then assessed MDAF's responsiveness to Lgmn, with the experimental design outlined in **Figure** **3**A. Briefly, monocytes were differentiated into foam cells using ox‐LDL, followed by incubation with MDAF or its control counterpart NAF (lacking the Lgmn‐responsive motif). Laser Confocal microscopy images (Figure 3B) and flow cytometry analysis (Figure 3C,D) revealed that, compared to the NAF group, MDAF was cleaved by Lgmn expressed in foamy BMDMs, resulting in a gradual increase in fluorescence intensity. Despite containing a self‐assembly motif, NAF lacks the Lgmn‐responsive motif, preventing cleavage‐mediated exposure of the self‐assembly domain and subsequent activation of AIE fluorescence. Identical results were observed in foamy RAW 264.7 cells, as depicted in Figure S7 (Supporting Information).

Building on this foundation, we further explored and validated whether MDAF can be controllably activated in response to the dynamic differentiation of monocytes. As illustrated in Figure 3E, our experimental design involved first enabling monocytes to internalize MDAF, followed by inducing their differentiation into foam cells using ox‐LDL to monitor whether MDAF undergoes gradual activation throughout this dynamic differentiation process. Before this, we investigated Lgmn expression during the dynamic differentiation of monocytes (Figure 3F), which revealed a differentiation‐dependent increase in Lgmn levels, with expression escalating alongside the extent of monocyte differentiation. Laser confocal microscopy images (Figure 3G) and flow cytometry analysis (Figure 3H,I) demonstrated that, in contrast to the PBS control group, MDAF was progressively activated during the dynamic differentiation of BMDMs into foam cells, with a concurrent gradual increase in fluorescence intensity. In the PBS group, by comparison, MDAF uptake by normal BMDMs remained unactivated. This phenomenon was recapitulated in dynamically differentiated RAW 264.7 cells (Figure S8, Supporting Information). Additionally, Figure 3J confirms that MDAF gradually assembles into nanofibers during the dynamic differentiation of BMDMs into foam cells.

### 2.4 Establishment and Evaluation of Apoe^−/−^ Mouse Model Harboring Both VAP and Stable Plaque

**Figure 4:**
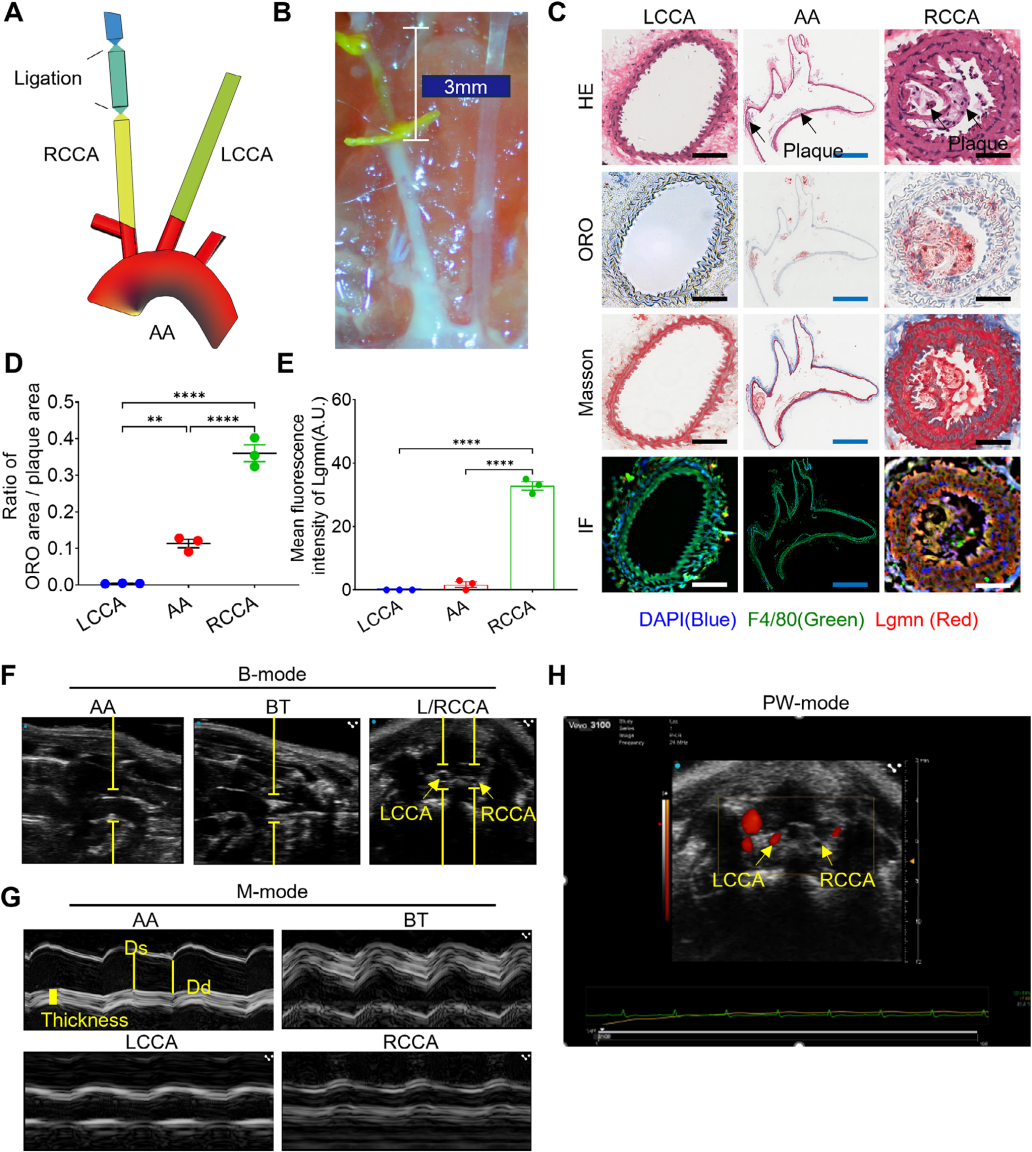
Establishment and evaluation of Apoe^−/−^ mouse model harboring both VAP and stable plaque. A,B) Schematic illustration of RCCA tandem stenosis surgery. C) Representative H&E, ORO, Masson staining, and IF images of the RCCA, LCCA, and AA of Apoe^−/−^mice. The scale bar (black and white) is 200 µm, and the scale bar (blue) is 2 mm. D,E) Quantitative analysis of ORO lipid and Lgmn mean fluorescence intensity (*n* = 3). F,G) B‐mode and M‐mode images of AA,^1^ BT, and L/RCCA in Apoe^−/−^ mice. (B) PW‐mode images of L/RCCA in Apoe^−/−^ mice. (*n* = 3). Data are shown as mean ± SD. Statistical analysis was performed by one‐way ANOVA.

To establish an Apoe^−/−^ mouse model harboring both VAP and stable plaques simultaneously, we performed a 3‐mm tandem stenosis surgery on the right common carotid artery (RCCA) of Apoe^−/−^ mice, as illustrated in **Figure** **4**A,B, to induce VAP formation in this specific region. Histological analyses (H&E, Oil Red O, Masson, and IF staining) presented in Figure 4C–E confirmed that, in contrast to the left common carotid artery (LCCA) and aortic arch (AA), the RCCA developed lipid‐rich, unstable plaques with high Lgmn expression. Additionally, we employed the same ultrasound imaging (USI) method of our previous work^[^ ^29^ ^]^ to further validate the effect of modeling. Results of B‐mode and M‐mode (Figure 4F,G) detected plaques and intimal thickening in the ascending aorta (AA^1^) and brachiocephalic trunk (BT) regions of the mice. The results of the PW‐mode analysis, presented in Figure 4H and Movie S1 (Supporting Information), further demonstrate that blood flow in the RCCA is barely detectable, likely attributable to vascular stenosis induced by the surgical procedure. In contrast, normal blood flow signals were consistently observed in the LCCA. These findings confirm that our surgical intervention selectively exerts a significant impact on plaque progression specifically within the RCCA, with minimal influence on the contralateral vessel.

### 2.5 In Vivo and Ex Vivo High Signal‐To‐Ratio Detection of VAP

**Figure 5:**
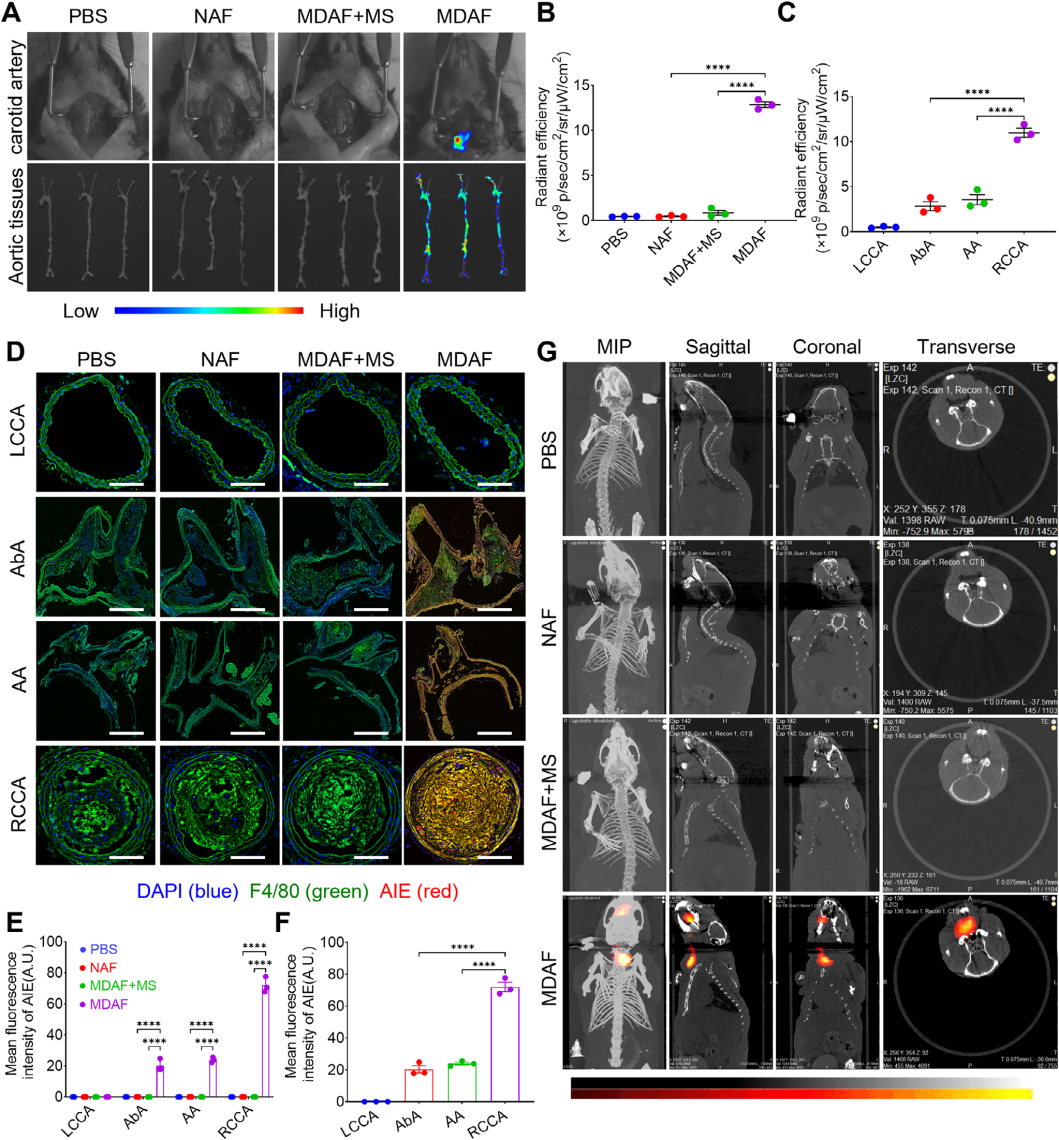
In vivo and ex vivo high signal‐to‐ratio detection of VAP. A) Fluorescence imaging of exposed carotid arteries in Apoe^−/−^ mice and their isolated aorta at 36 h after treatment in each group. (AIE dose, 0.8 mg kg^−1^) (*n* = 3). B) Quantitative analysis of fluorescence signals among different treatment groups (*n* = 3). C) Quantitative comparative analysis of fluorescence signals in LCCA, abdominal aorta (AbA), aortic arch (AA), and RCCA in the MDAF group. (*n* = 3). D) IF sections of LCCA, abdominal aorta (AbA), aortic arch (AA), and RCCA at 36 h after treatment in each group. (*n* = 3). The scale bar (white) is 100 µm. E) Quantitative analysis of mean fluorescence intensity among different treatment groups (*n* = 3). F) Quantitative comparative analysis of mean fluorescence intensity in LCCA, AbA, AA, and RCCA in the MDAF group. (*n* = 3). G) High signal‐to‐ratio FLECT imaging at 36 h after treatment in each group. (*n* = 3). Data are shown as mean ± SD. Statistical analysis was performed by one‐way and two‐way ANOVA.

Subsequently, we evaluated the efficacy of the MDAF for VAP detection in Apoe^−/−^ mice. First, in vivo fluorescence imaging (Figure S9A,B, Supporting Information) revealed that MDAF was efficiently enriched in the neck of Apoe^−/−^ mice within 48 h, with fluorescence intensity peaking at 36 h, reaching 3.54‐fold that of the MDAF + monocyte scavenger (MS) group. MS can be used to eliminate monocytes in mice. Therefore, we utilized it to observe and verify that monocytes play a decisive role in MDAF targeting VAP and its signal activation in vivo. To further confirm whether MDAF specifically accumulates in VAP of RCCA and achieves controllable signal activation, we exposed the neck skin of mice and performed fluorescence imaging of carotid arteries. As shown in Figure S9C,D (Supporting Information), MDAF successfully achieved specific enrichment and fluorescence activation in VAP of RCCA. To verify the specificity of MDAF distribution and activation in VAP, we harvested the entire aorta from probe‐treated mice at 36 h for ex vivo imaging (**Figure** **5**A). MDAF was distributed and activated not only in the RCCA but also in the aortic arch (AA) and abdominal aorta (AbA), with no detectable signal in the LCCA—consistent with the mouse modeling results in Figure 4. Quantitative analysis (Figure 5B,C) showed that MDAF fluorescence intensity in the RCCA was 3.09‐fold higher than in the AA and 3.88‐fold higher than in the AbA. Further isolation of arteries from these regions and IF sections (Figure 5D–F) confirmed the specific distribution and activation of MDAF in RCCA VAP at the microscopic level, with an average fluorescence intensity 3.01‐fold higher than in the AA and 3.55‐fold higher than in the AbA.

To further enhance the SNR of the probe, after treatment for 36 h, FLECT imaging was performed to scan the region from the head to the chest (≈30 mm) of the Apoe^−/−^ mice. The results are presented in Figure 5G and Movie S2 (Supporting Information). MDAF specifically accumulated in the VAP at the RCCA and activated its fluorescence signal, whereas no distribution or activation was observed at the LCCA. Ex vivo imaging and quantitative analysis of the mouse organs were subsequently conducted, as shown in Figure S10 (Supporting Information). Although inevitable probe aggregation occurred in metabolic organs, such as the liver and kidney, the average fluorescence intensity in arteries was 4.71‐fold higher than that in the liver when compared to overall isolated arterial tissue, demonstrating the probe's excellent SNR. Additionally, biosafety data in Figure S11 (Supporting Information) show that probes and treatment from all groups exhibited no apparent toxicity to blood or organ tissues.

### 2.6 Ex Vivo Imaging of the Human Atherosclerotic Intima of MDAF

**Figure 6:**
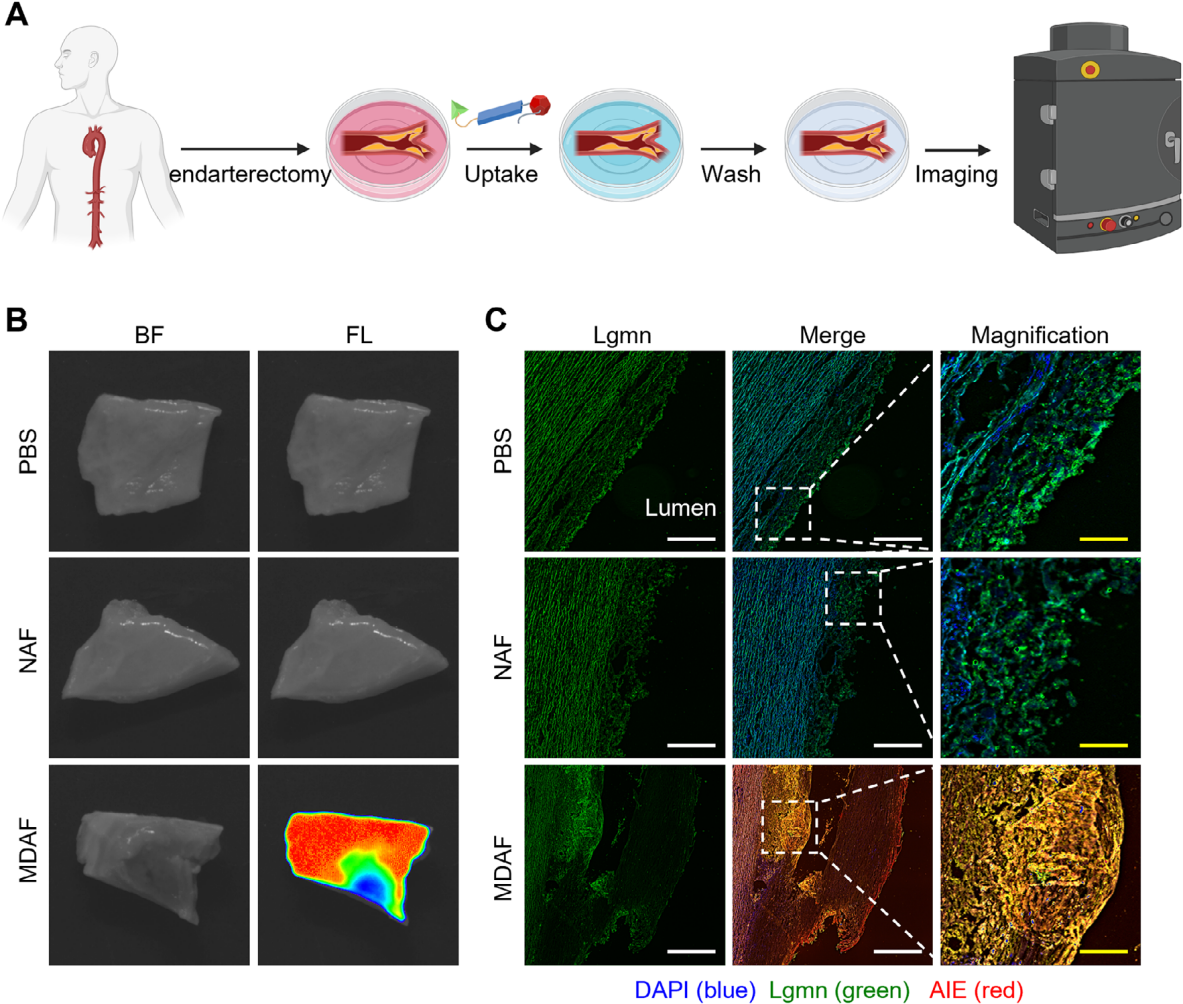
Ex vivo imaging of the human atherosclerotic intima of MDAF. A) Schematic illustration of the imaging procedure after incubating the isolated human atherosclerotic intima with MDAF (AIE concentration, 5 µm). B,C) Ex vivo fluorescence imaging of the human atherosclerotic intima in three patients and the corresponding IF sections. The scale bar (white) is 200 µm, and the scale bar (yellow) is 100 µm.

Additionally, we successfully obtained clinical specimens of the ascending aortic intima from three patients with advanced atherosclerosis, which were used to validate the efficacy and translational potential of MDAF for detecting human plaques. As illustrated in **Figure** **6**A, human aortic intima samples were co‐incubated with MDAF or NAF (negative control probe), followed by probe removal and ex vivo imaging. Figure 6B demonstrates that MDAF effectively penetrated the aortic intima with appreciable thickness and triggered fluorescence activation, whereas NAF failed to elicit fluorescence signals. IF analysis in Figure 6C further revealed successful co‐localization of MDAF with Lgmn within atherosclerotic plaques, highlighting its translational potential and clinical utility for human plaque diagnosis.

## 3 Conclusion

Overall, in recent years, targeted molecular imaging probes designed based on the VAP microenvironment have achieved considerable progress.^[^ ^12^, ^33^, ^34^, ^35^ ^]^ However, challenges such as off‐target effects and insufficient signal‐to‐noise ratios (SNR) still persist.^[^ ^9^, ^10^, ^11^ ^]^ Furthermore, the complex nature of the VAP microenvironment renders probes targeting a single biomarker incapable of comprehensively reflecting plaque vulnerability.^[^ ^19^, ^20^, ^21^ ^]^ Cell‐mediated delivery strategies represent an emerging research direction in the diagnosis and treatment of atherosclerotic plaques.^[^ ^28^, ^29^ ^]^ Monocytes, endowed with natural chemotaxis toward plaques and intrinsic “immune scouting” capabilities to sense VAP vulnerability, have emerged as ideal probe carriers^[^ ^26^, ^27^ ^].^ Inspired by this, we designed MDAF, which functions as a “scout” when hitchhiked on monocytes, successfully traversing the VAP physiological barrier and achieving effective accumulation within VAP lesions.

MDAF enables spatiotemporally controlled activation of imaging signals through the dynamic differentiation and activation of monocytes, effectively reducing probe activation in non‐pathological regions. Notably, it can distinguish VAP from stable plaques in the same Apoe^−/−^ mice, significantly improving both the SNR and spatiotemporal resolution of VAP detection. Furthermore, our findings demonstrate that MDAF exhibits high specificity and sensitivity in detecting clinical specimens of the ascending aortic intima from patients with severe atherosclerosis, validating its potential for clinical translation. This study not only advances cell‐mediated delivery strategies in VAP diagnosis but also provides novel insights for the development of efficient, intelligent, and controllable molecular probes to address key challenges in VAP detection. Additionally, it offers a reference framework for the design of imaging probes for other disease contexts.

## 4 Experimental Section

The detailed experiments are available in the Supporting Information.

## Conflict of Interest

The authors declare no conflict of interest.

## Supporting information



Supporting Information

Supplemental Movie 1

Supplemental Movie 2

## Data Availability

The data that support the findings of this study are available from the corresponding author upon reasonable request.
