# Supplementary material for: Monocyte‐Differentiation‐Activated Fluorescent “Scout” Probe for Precise in Vivo Detection of Vulnerable Plaque
Source: Adv Sci (Weinh). 2025 Dec 2;13(9):e15289. doi: 10.1002/advs.202515289 (PMC12903971; doi:10.1002/advs.202515289)
Supplement: Supplementary file 1 — Supporting Information [file ADVS-13-e15289-s001.docx]

Supporting Information

**Monocyte-Differentiation-Activated Fluorescent “Scout” Probe for Precise *In Vivo* Detection of Vulnerable Plaque**

*Zechuan Li, Jiankai Dong, Zhengkun Liu, Chaoke Zhang, Jisen Li, Ying Tao, Ding Yang, Yansong Liu, Haoting Chen, Lu Liu, Jingsen Ji, Feng Cao, Dan Ding, Qian Liu*, Chenxing Fu*, Weisheng Guo**

Z. Li, Z. Liu, Y. Tao, Y. Liu, H. Chen, L. Liu, J. Ji, C. Fu, W. Guo

Department of Cardiology, Guangzhou Institute of Cardiovascular Disease, Guangdong Key Laboratory of Vascular Diseases, The Second Affiliated Hospital, School of Biomedical Engineering, Guangzhou Medical University, Guangzhou 510260, China

E-mail: [guo_wei_sheng@gzhmu.edu.cn](mailto:guo_wei_sheng@gzhmu.edu.cn); [fuchenxing@gzhmu.edu.cn](mailto:fuchenxing@gzhmu.edu.cn).

Q. Liu

Department of Urology, Tianjin First Central Hospital, Tianjin 300192, China

Email: [simonlq@163.com](mailto:simonlq@163.com)

J. Dong

Department of Cardiology, The First Affiliated Hospital of Bengbu Medical University, Bengbu, 233000, China

J. Li, D. Ding

Key Laboratory of Bioactive Materials for the Ministry of Education, College of Life Sciences, Nankai University, Tianjin 300071, China

D. Yang

Department of Radiology, Peking University Cancer Hospital & Institute, Key Laboratory of Carcinogenesis and Translational Research (Ministry of Education/Beijing), Beijing 100142, China

Chaoke Zhang

School of Materials Science and Engineering, University of Jinan, Jinan 250022, China

L. Liu

Nanomedicine Research Center, The Third Affiliated Hospital of Sun Yat-sen University, Guangzhou, 510630, China

F. Cao

National Clinical Research Center for Geriatric Diseases, The Second Medical Center

Chinese PLA General Hospital, Beijing 100853, China

**Keywords:** atherosclerosis, molecular imaging, cell-mediated delivery, monocyte dynamic differentiation, aggregation-induced emission

**Experimental Section**

*Materials*: The peptides applied in this study were synthesized and provided by Hubei Qiangyao Biotechnology Co., Ltd. The AIE molecules were provided by Professor Ding Dan's team from Nankai University, Tianjin. Cy5.5-Mal was purchased from Shanghai Mindray Biochemical Technology Co., Ltd. Ox-LDL was supplied by Guangzhou Yiyuan Biotechnology Co., Ltd. The monocyte scavenger (MS) agent was obtained from Shanghai Yisheng Biotechnology Co., Ltd., China. All flow cytometry antibodies were procured from BD Company, USA.

*Experimental Animals*: Male Apoe^⁻/⁻^ mice (6-8 weeks old) were purchased from Cyagen Biosciences, Inc. (Jiangsu, China). All mice were fed in a clean laboratory animal facility with free access to food and water at Guangzhou Medical University. All experimental procedures were approved by the Institutional Animal Care and Use Committee of Guangzhou Medical University and conducted in accordance with their guidelines (Acceptance number: GY-2023-531).

*Synthesis of Fluorescent Probes*: Fluorescent probes were synthesized by peptides (MDAP, NAP, SAP) and AIE molecules with a molar ratio of 1:1.1−1.2 through the Michael addition reaction. The components were mixed and dissolved in an equal volume mixture of DMF and deionized water, stirring thoroughly and reacting overnight, then dialysis was performed to remove excess peptides and AIE molecules. The purified samples were freeze-dried. Post-freezing, the AIE-labeled probes were redissolved in ACN. An ACN solution and deionized water, both containing 0.1% TFA, were prepared as the organic and aqueous phases for HPLC analysis. HPLC was used to verify the conjugation between peptide molecules and AIE fluorescent probes, as well as product purity. Molecular weights were determined by high-resolution mass spectrometry and MALDI-TOF mass spectrometry.

*Characterization of the Self-assembly Function of MDAF*: MDAF and SAF solutions (1 μM) were prepared. Ten microliters of each sample were dropped onto a copper mesh, air-dried overnight, and stained with 3% uranyl acetate for 3 minutes. The excess stain was removed, and samples were air-dried again before capturing TEM images to observe nanofiber morphology. Additionally, solutions were freeze-dried, mixed with KBr at a sample: KBr mass ratio of 1:200, and characterized by FTIR. In addition, MDAF was incubated with recombinant Lgmn in a working buffer at 37 °C for 15 min, 30 min, 1 h, 2 h, and 6 h. TEM samples were prepared from reaction solutions to verify Lgmn-mediated MDAF cleavage and self-assembly-induced nanofiber formation. HPLC assisted in confirming MDAF cleavage, while UV-fluorescence spectrophotometry detected AIE molecule fluorescence intensity.

*In Vitro Targeting Efficiency of MDAP-Cy5.5 on Monocytes:* BMDMs from 6-8 weeks C57 mice and the monocyte/macrophage cell line RAW 264.7 were used to simulate circulating monocytes *in vitro*. MDAP-Cy5.5 and Cy5.5 (Cy5.5 concentration, 5 μM) were co-incubated with monocytes for 1 h. Confocal microscopy and flow cytometry were used to compare their *in vitro* targeting efficiency.

*In Vivo Targeting Efficiency of MDAP-Cy5.5 on Inflammatory Monocytes:* Apoe^⁻/⁻^ mice were fed a Western diet for 12 weeks, then divided into two groups. MDAP-Cy5.5 or free Cy5.5 (Cy5.5 dose, 0.8 mg/kg) was injected intravenously. Targeting efficiency for inflammatory monocytes and uptake by other blood leukocytes were analyzed at 2 and 6 hours post-administration. Whole blood was collected, lysed, and treated with Anti-mouse CD16/CD32 mAb to block Fc receptors. Antibodies (APC/Cyanine7 anti-mouse Ly-6C, FITC anti-mouse CD11c, PerCP/Cyanine5.5 anti-mouse/human CD11b, PE anti-mouse Ly-6G/Ly-6C (Gr-1)) were added at a 1:100 (v/v) ratio, incubated on ice for 30 min, fixed, and analyzed by flow cytometry using Cyt Expert software.

*Lgmn Expression Induced by Monocyte Differentiation*: BMDMs and RAW 264.7 cells were incubated with ox-LDL (0, 20, 40, 60, 80 μg/mL) for 48 h to induce foam cell differentiation. RNA was extracted for qPCR to detect Lgmn mRNA expression. For dynamic differentiation analysis, BMDMs and RAW 264.7 cells were treated with 80 μg/mL ox-LDL, harvested at 3, 6, 12, 24, 48 h, and qPCR analyzed Lgmn mRNA changes. Primer sequences for Lgmn:

- Forward: TGGACGATCCCGAGGATGG

- Reverse: GTGGATGATCTGGTAGGCGT

*Lgmn-Responsive Activation of MDAF*: BMDMs and RAW 264.7 cells were differentiated into foam cells with 80 μg/mL ox-LDL for 48 h, then co-incubated with MDAF or NAF solutions (AIE concentration, 5 μM) for 1 h. After removing excess probes, cells were incubated in a medium for 0, 0.5, 1, 2, 4, and 6 h, fixed, and stained with DAPI. Laser confocal microscopy and flow cytometry were used to observe fluorescence activation and quantify fluorescence intensity.

*Dynamic Activation of MDAF During Monocyte Differentiation*: BMDMs and RAW 264.7 cells were first incubated with MDAF (AIE concentration, 5 μM) for 1 h, then were washed and incubated with 80 μg/mL ox-LDL for 0, 3, 6, 12, 24, 48 h. After fixation and DAPI staining, laser confocal microscopy and flow cytometry evaluated MDAF fluorescence activation during differentiation. Bio-TEM samples of foam cells were prepared to observe intracellular nanofibers.

*Evaluation of the Modelling Effect of* Apoe^⁻/⁻^ *Mice*: 8-week-old Apoe^⁻/⁻^ mice were fed a Western diet for 4 weeks, followed by RCCA stenosis surgery to induce VAP. Post-surgery, penicillin was administered, and mice were fed a Western diet for 8 weeks. The whole aorta, including RCCA, LCCA, and AA, was harvested for frozen sections. Plaque vulnerability was evaluated by H&E, Masson, ORO staining, IF, and high-resolution ultrasound imaging (USI). The sections were scanned and quantified by Image J.

USI analyzed the ascending aorta (AA^1^), brachiocephalic trunk (BT), RCCA, and LCCA. Mice were anesthetized, depilated, and placed on a temperature-controlled board. Conductive glue was applied, and heart rate/respiratory rate was monitored. The USI probe was fixed, and an ultrasonic coupling agent was applied. B, M, and PW modes were used to measure vascular wall thickness, arterial elasticity (Ds−Dd), and RCCA/LCCA blood flow velocities.

*In Vivo Fluorescence Imaging*: Mice were depilated, fasted, and grouped (PBS, NAF, MDAF+MS, MDAF; 0.8 mg/kg AIE, intravenously injected). MDAF + MS group received 100 μL MS intraperitoneally. Imaging was performed at 1, 12, 24, 36, 48 h. To reduce non-specific signals, carotid arteries were exposed post-anesthesia, and imaging was repeated at 12, 24, 36, and 48 h. Fluorescence was analyzed by Living Image software.

Fluorescence Emission Computed Tomography (FLECT): Mice were depilated, fasted, and injected as above. FLECT imaging of the neck was conducted 36 h later. CT imaging was first performed, followed by 3D FLECT scanning (from head to chest about 30 mm, 1 mm per layer). Data were 3D-reconstructed for FLECT/CT analysis using Insyte FLECT/CT.

*Isolated Tissue and Organ Fluorescence Imaging*: After *in vivo* imaging, mice were anesthetized, and the whole aorta from the carotid artery to the iliac artery and major organs were dissected. Excess fat was removed, and tissues were imaged by Living Image. Aorta segments (RCCA, LCCA, AA, AbA) were frozen for IF staining, scanned by DMi8 microscope, and quantified by Image J.

*Ex Vivo Imaging of Human Atherosclerotic Aortic Intima*: All experimental samples were required from The Second Affiliated Hospital of Guangzhou Medical University with the ethical approval (Acceptance number: B2023-033). Samples were incubated with PBS, NAF, or MDAF (AIE concentration, 5 μM) for 1 h, washed, imaged, fixed, and prepared for IF sections.

*Biosafety Assessment*: Major organs were fixed for H&E staining, and serum (stored at −80°C) and whole blood (4°C) were analyzed for biochemical (ALT, AST, BUN, UA) and hematological (monocyte count, WBC, RBC) parameters.

**Statistical Analysis:** Continuous data were presented as mean ± standard deviation (SD), and were tested for normality using the D’Agostino-Pearson or Shapiro-Wilk test. For multiple comparisons, data were analyzed using ANOVA followed by Tukey post hoc tests. The measurements were taken from distinct samples. A significant difference was considered when the P value was less than 0.05. Statistical analysis was conducted using GraphPad Prism 9.5 software.

Supplementary Figures


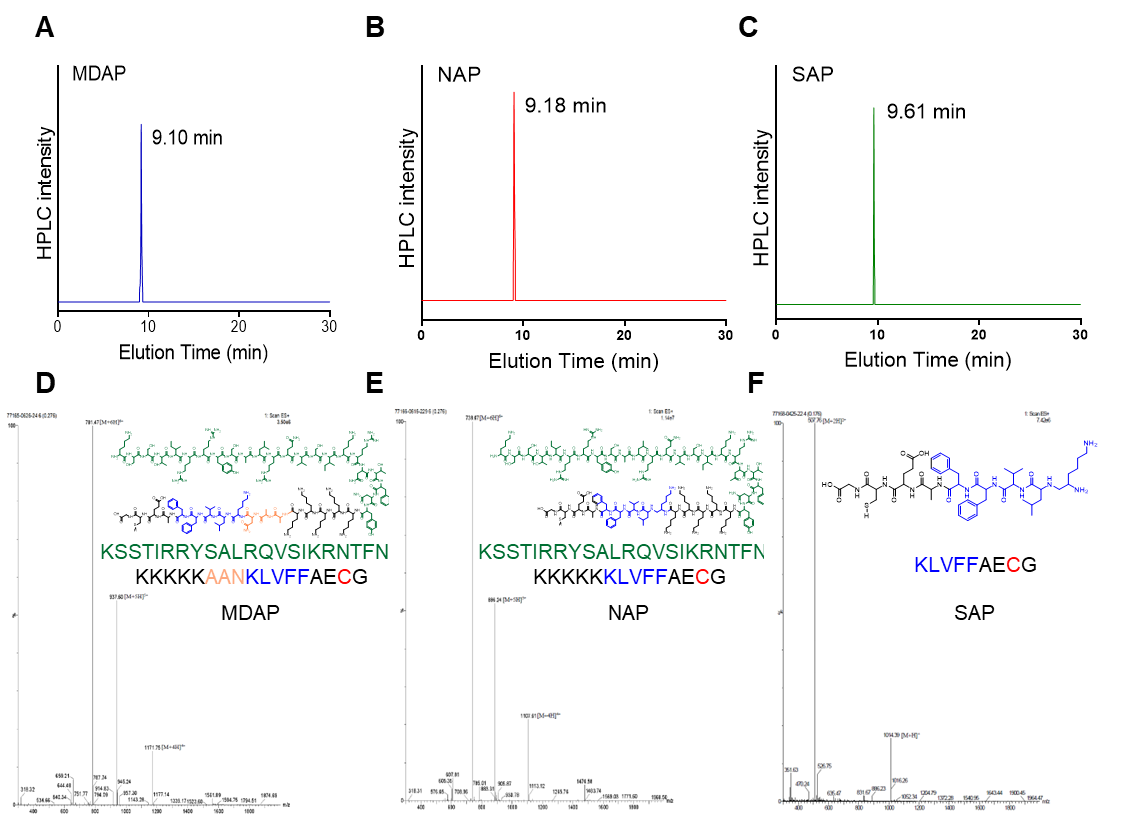


**Figure S1.** HPLC and MALDI-TOF analysis of peptides. (A-C) HPLC analysis of MDAP, NAP, and SAP. (D-F) High-resolution mass spectra of MDAP, NAP, and SAP.


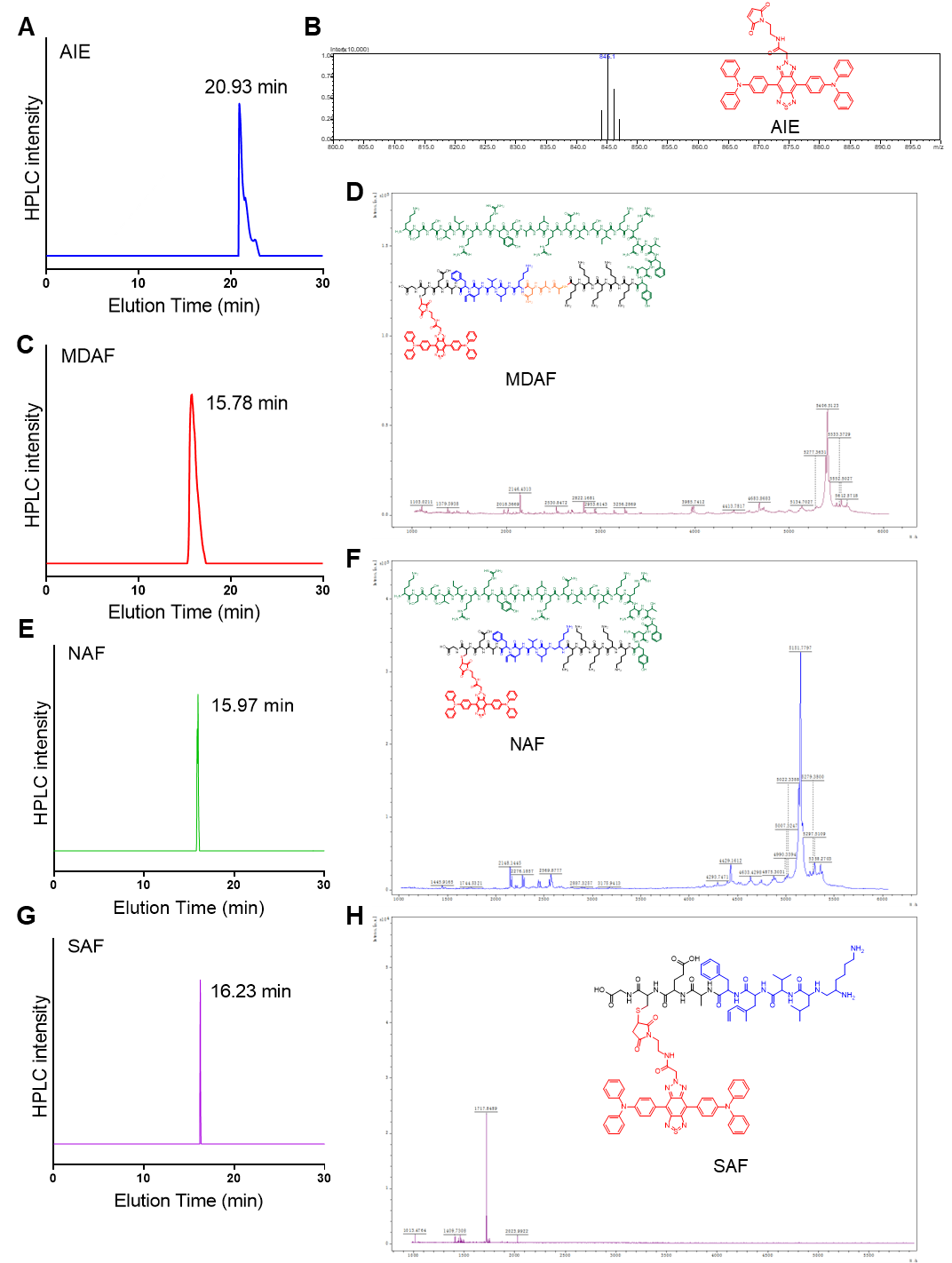


**Figure S2.** Synthesis and characterization of fluorescent probes. (A, B) HPLC analysis and mass spectra of the AIE molecule. (C, D) HPLC analysis and MALDI-TOF mass spectra of MDAF. (E, F) HPLC analysis and MALDI-TOF mass spectra of NAF. (G, H) HPLC analysis and MALDI-TOF mass spectra of SAF.


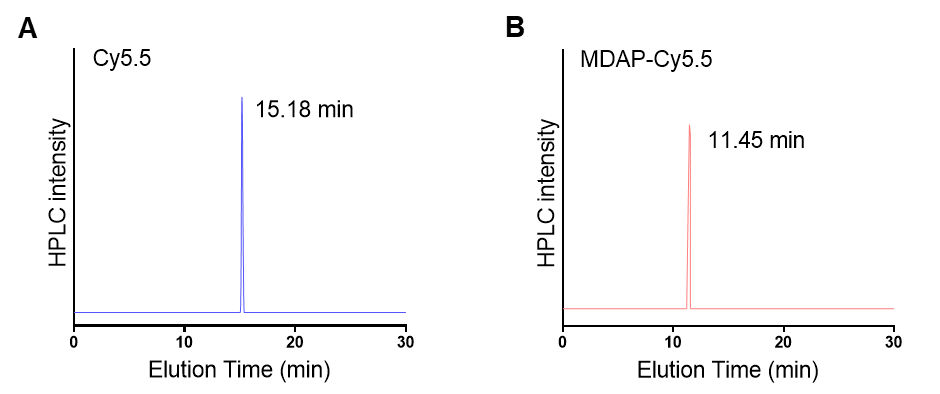


**Figure S3.** HPLC analysis of Cy5.5 and MDAP-Cy5.5.


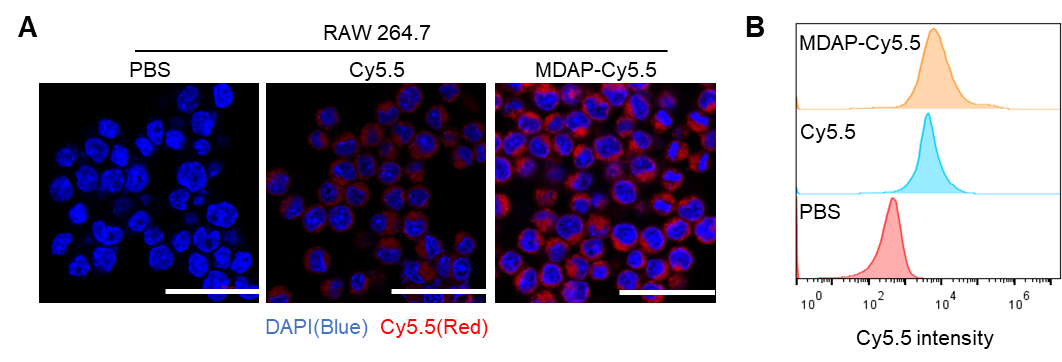


**Figure S4.** Specific targeting of MDAP-Cy5.5 to RAW 264.7 cells. (A, B) Laser confocal microscopy images and flow cytometry quantitative analysis of Cy5.5 and MDAP-Cy5.5 (Cy5.5 concentration, 5 μM) uptake by RAW 264.7 cells. (*n* = 3). The scale bar (white) is 50 μm.


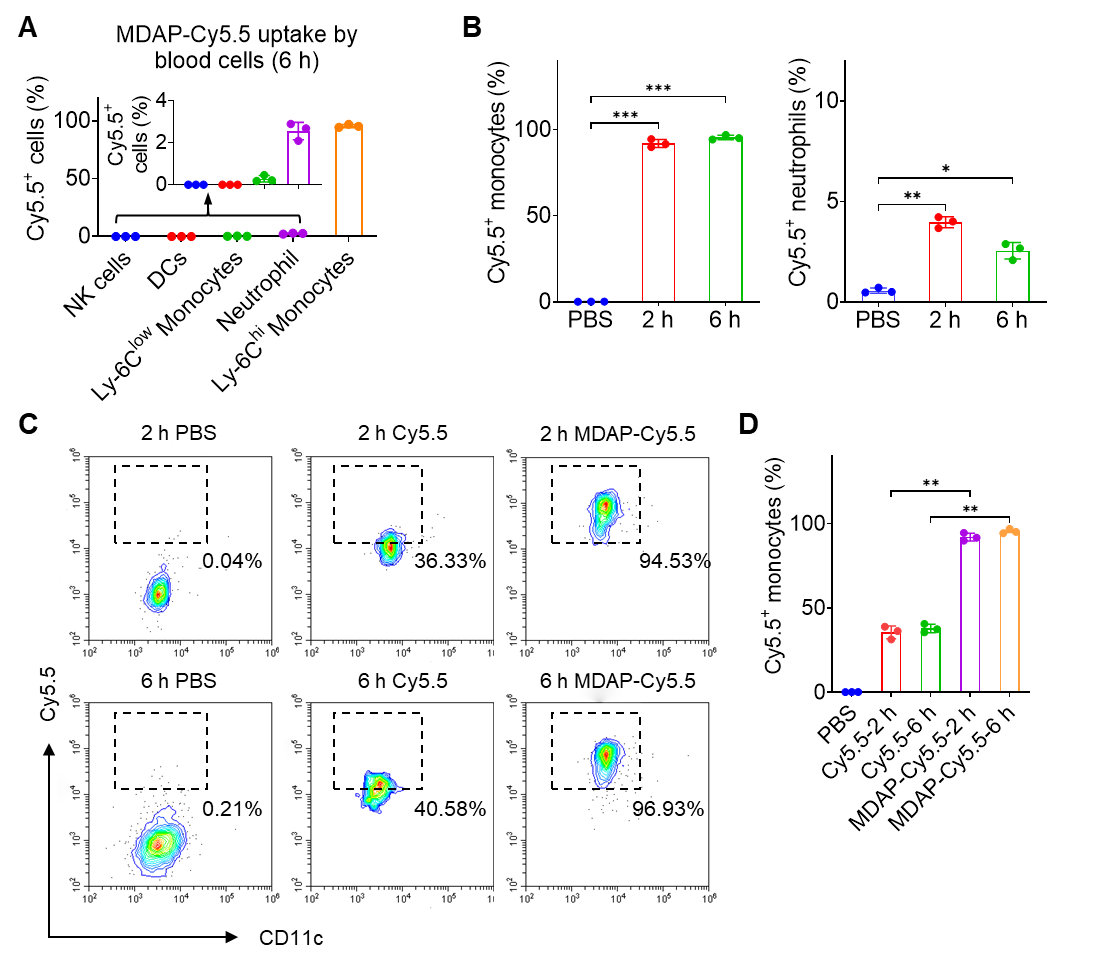


**Figure S5.** Specific targeting of MDAP-Cy5.5 to inflammatory monocytes. (A) Uptake proportions of MDAP-Cy5.5 (Cy5.5 dose, 0.8 mg/kg) by different blood leukocyte subsets at 6 h. (B) Uptake proportions of MDAP-Cy5.5 (Cy5.5 dose, 0.8 mg/kg) by monocytes and neutrophils at 2 and 6 h, respectively (*n* = 3). (C, D) Uptake efficiency comparison of Cy5.5 and MDAP-Cy5.5 (Cy5.5 dose, 0.8 mg/kg) by blood inflammatory monocytes and their quantitative analysis (*n* = 3). Data are shown as mean ± SD. Statistical analysis was performed by one-way ANOVA.


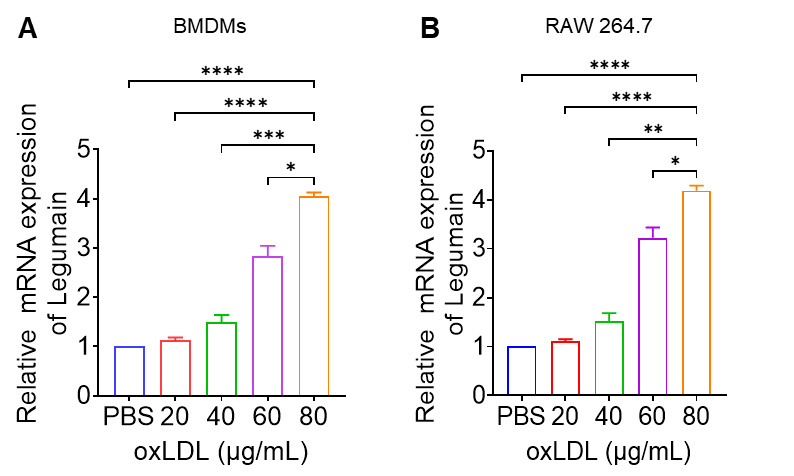


**Figure S6.** Upregulated legumain expression along with elevated ox-LDL concentration. (A, B) Changes of Lgmn mRNA expression with ox-LDL concentration in BMDMs and RAW 264.7 cells (*n* = 3). Data are shown as mean ± SD. Statistical analysis was performed by one-way ANOVA.


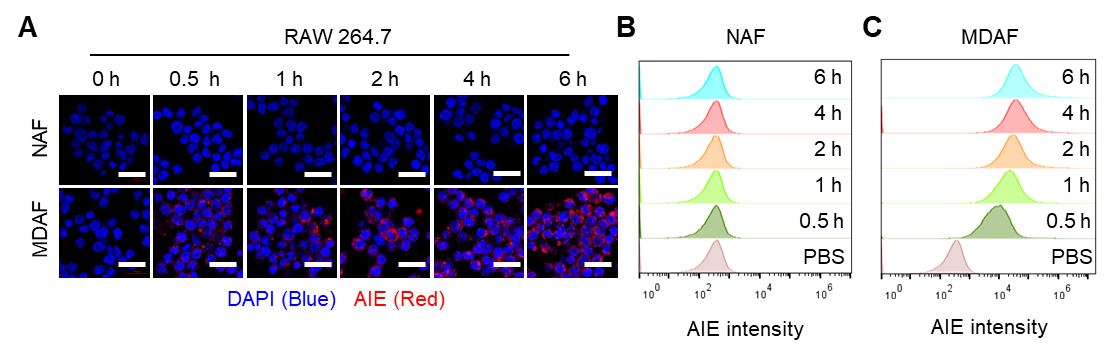


**Figure S7.** Lgmn responsive activation of MDAF in foamy RAW 264.7 cells. (A-C) Laser confocal microscopy images and flow cytometry quantitative analysis of Lgmn-responsive activation of MDAF and NAF in foamy RAW 264.7 cells. (*n* = 3). The scale bar (white) is 20 μm.


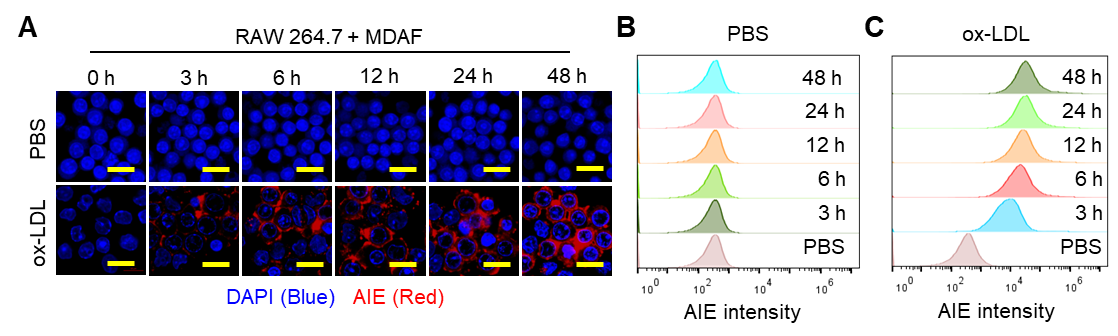


**Figure S8.** Monocyte differentiation-responsive activation of MDAF in normal and foamy RAW 264.7 cells. (A-C) Laser confocal microscopy images and flow cytometry quantitative analysis of the fluorescence intensity of activated MDAF in normal and differentiating RAW 264.7 cells. (*n* = 3). The scale bar (yellow) is 10 μm.


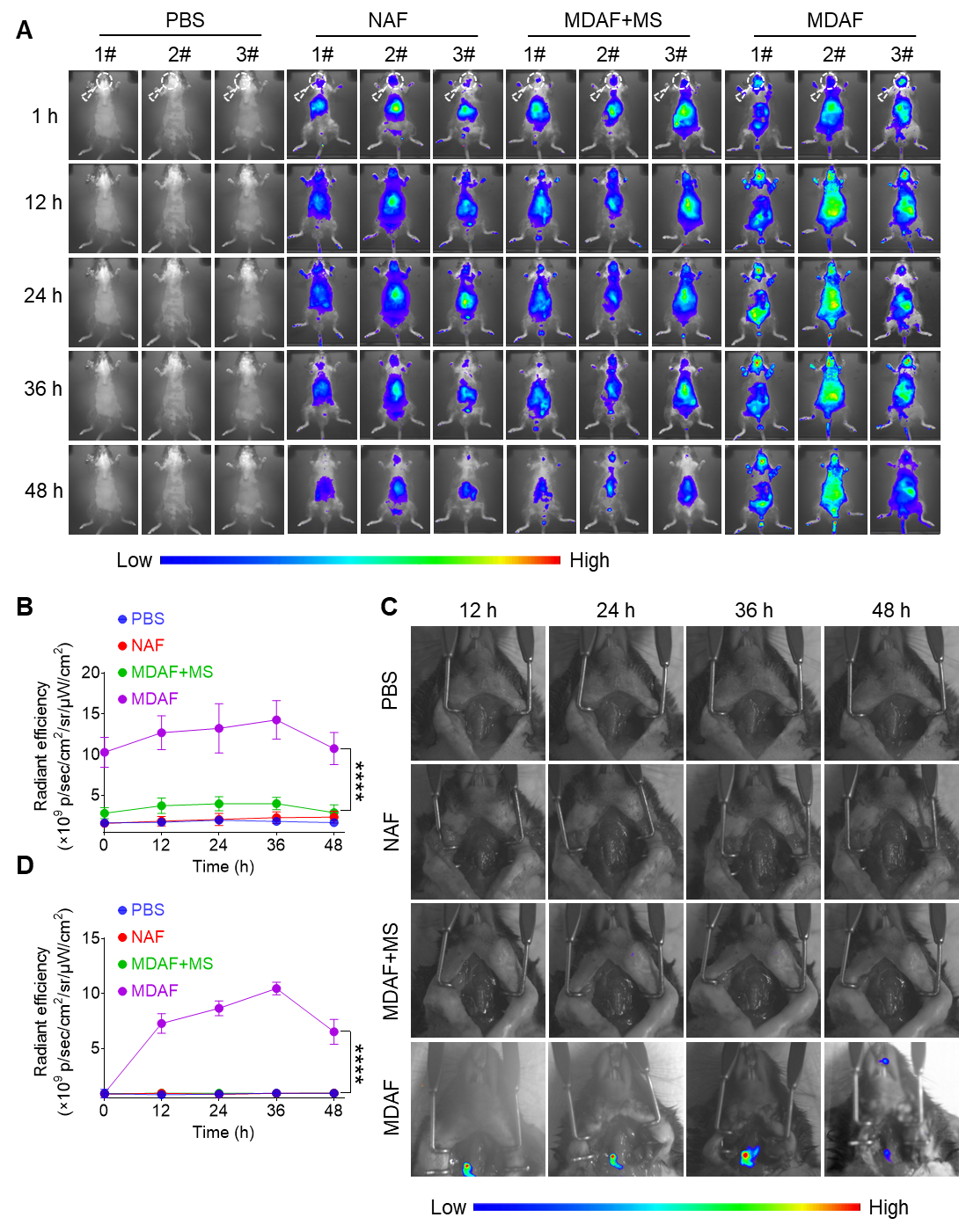


**Figure S9.** *In vivo* fluorescence imaging of Apoe^⁻/⁻^ mice. (A, B) *In vivo* fluorescence imaging of Apoe^⁻/⁻^ mice at different time points and their quantitative analysis. (C, D) Fluorescence imaging of exposed carotid arteries in Apoe^⁻/⁻^ mice at different time points and their quantitative analysis. (*n* = 3). Data are shown as mean ± SD. Statistical analysis was performed by two-way ANOVA.


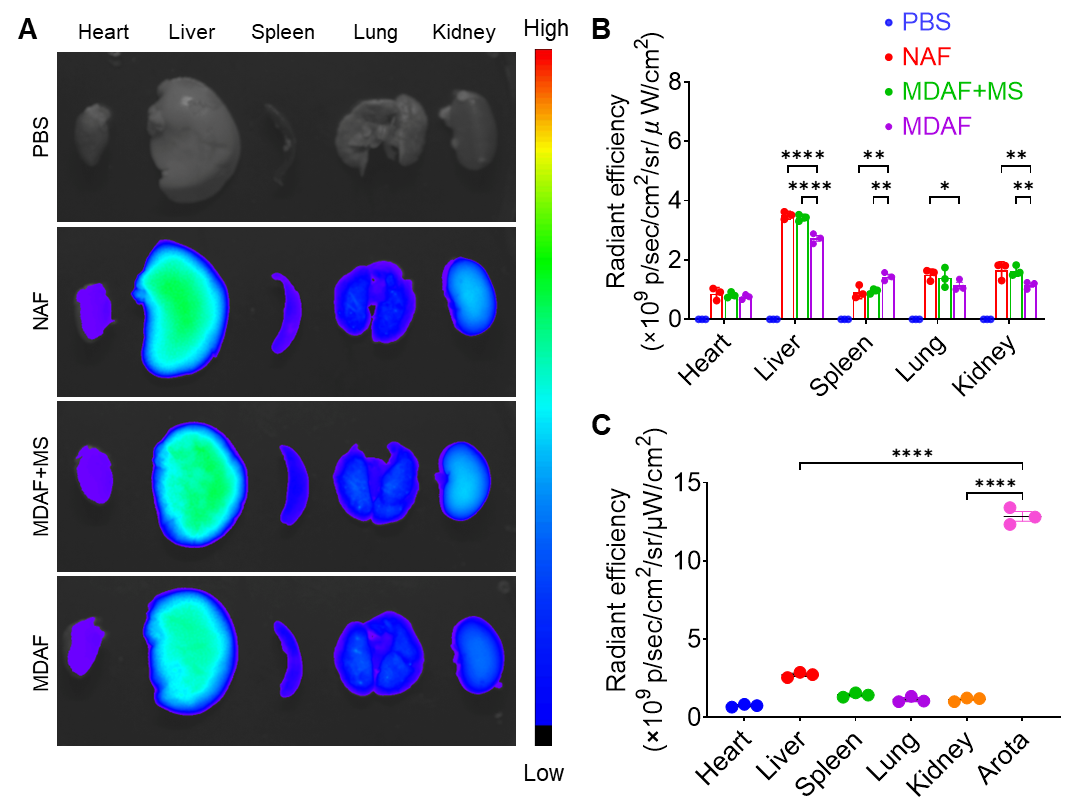


**Figure S10.** *Ex vivo* fluorescent imaging of isolated organs of Apoe^⁻/⁻^ mice. (A, B) Fluorescence imaging and quantitative analysis of isolated organs of Apoe^⁻/⁻^ mice at 36 hours after treatment. (C) Quantitative comparative analysis of isolated organs and isolated arterial tissues of Apoe^⁻/⁻^ mice at 36 hours after treatment (*n* = 3). Data are shown as mean ± SD. Statistical analysis was performed by one-way and two-way ANOVA.


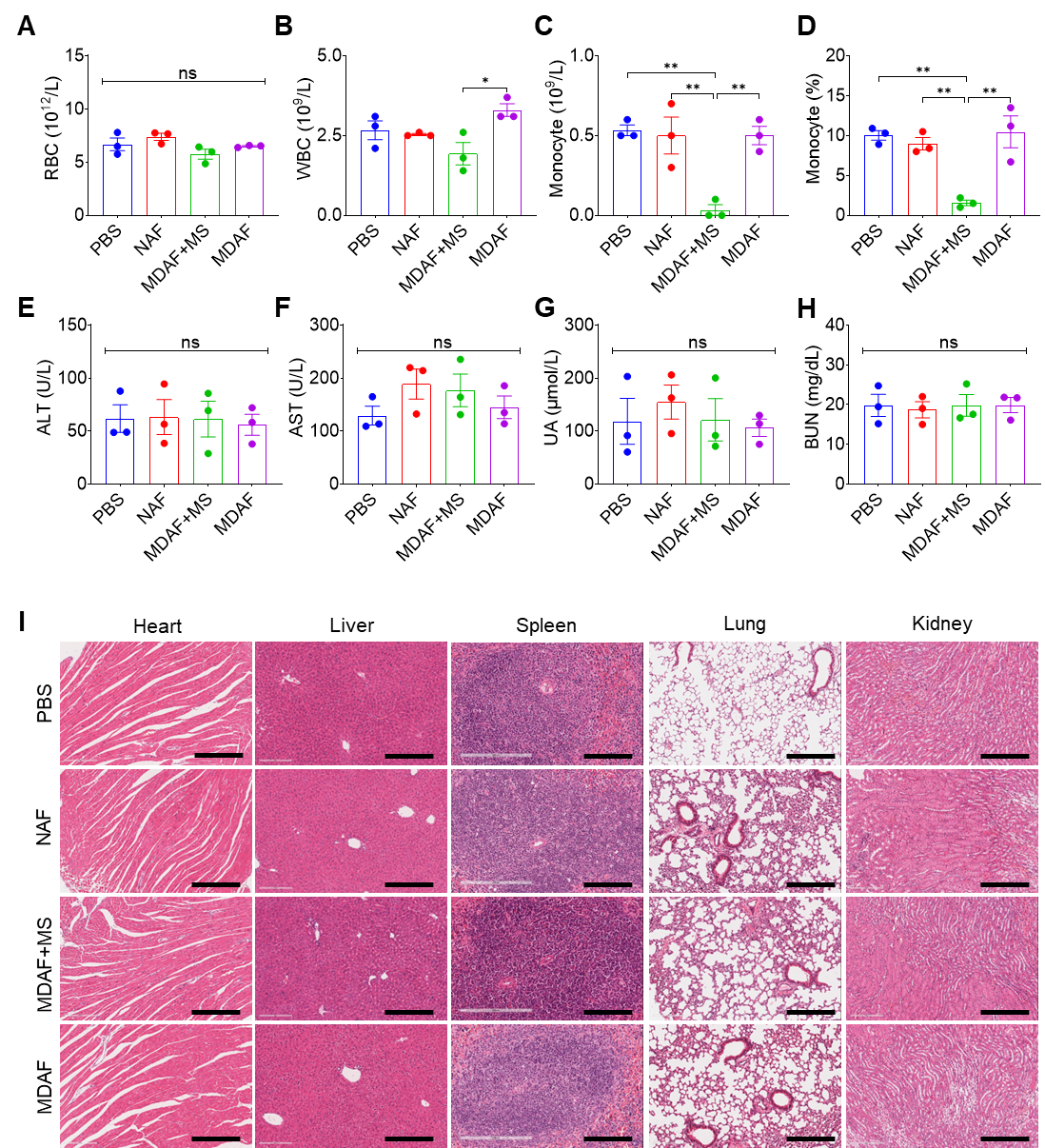


**Figure S11.** *In vivo* biosafety by blood and pathological evaluation. (A-D) Safety evaluation by blood routine index and (E-H) blood biochemical index after treatment. (I) Representative H&E staining images of typical organs in PBS, NAF, MDAF + MS, and MDAF groups (*n* = 3). The scale bar (black) is 200 μm. Data are shown as mean ± SD. Statistical analysis was performed by one-way ANOVA.
